# World Health Organization High Priority Pathogens: Ophthalmic Disease Findings and Vision Health Perspectives

**DOI:** 10.3390/pathogens10040442

**Published:** 2021-04-08

**Authors:** Sanjana Kuthyar, Casey L. Anthony, Tolulope Fashina, Steven Yeh, Jessica G. Shantha

**Affiliations:** 1 Emory Eye Center, Department of Ophthalmology, Emory University School of Medicine, Atlanta, GA 30322, USA; sanjana.kuthyar@emory.edu (S.K.); casey.leigh.anthony@emory.edu (C.L.A.); tfashin@emory.edu (T.F.); 2 Department of Ophthalmology, Truhlsen Eye Institute, University of Nebraska Medical Center, Omaha, NE 68105, USA

**Keywords:** emerging infectious disease, ophthalmic manifestations, ophthalmic sequalae, retinitis, uveitis, viral persistence, ebola virus disease, ebola survivor, marburg virus disease, anterior uveitis, posterior uveitis, tear film transmission, COVID-19

## Abstract

Recent Ebola epidemics, the ongoing COVID-19 pandemic, and emerging infectious disease threats have highlighted the importance of global infectious diseases and responses to public health emergencies. Ophthalmologists are essential health care workers who provide urgent and emergent vision care services during outbreaks and address the ocular consequences of epidemic and pandemic infectious diseases. In 2017, the World Health Organization (WHO) identified high priority pathogens likely to cause a future epidemic with the goal of guiding research and development to improve diagnostic tests, vaccines, and medicines. These measures were necessary to better inform and respond to public health emergencies. Given the ocular complications associated with emerging infectious diseases, there is a need to recognize the ophthalmic sequelae for future vision health preparedness for potential future outbreaks. The WHO High Priority pathogens list provides a roadmap for ophthalmologists and subspecialty providers that will guide strategic areas of research for clinical care and preparedness for future pandemic threats. This review summarizes these key viral pathogens, summarizes major systemic disease findings, and delineates relevant ocular complications of the WHO High Priority pathogens list, including Crimean-Congo hemorrhagic fever, Filovirus diseases (Ebola virus disease and Marburg hemorrhagic fever), human Coronaviruses, Lassa Fever, Nipah virus infection, Zika, and Rift Valley fever.

## 1. Introduction

In response to the West African Ebola virus disease (EVD) epidemic in 2014–2016, experts convened by the World Health Organization (WHO) identified a list of eight emerging infectious diseases with the potential to evolve into a public health emergency in the future, and for which there were few countermeasures for a sufficient preventive or curative response [1,2]. These WHO efforts aimed to better identify country-specific constraints in response capabilities, generate accelerated research and development, and enhance the WHO’s capacity to facilitate access to diagnostic, preventive, and therapeutic products in public health emergencies. The diseases included: Crimean-Congo hemorrhagic fever, Filovirus diseases (Marburg hemorrhagic fever and EVD), human Coronaviruses, Lassa Fever, Nipah virus infection, Zika, Rift Valley fever, and Disease X [2].

New infectious agents are often underestimated, and rapid public health recognition and interventions are necessary to reduce further infection. Ophthalmologists remain essential to the efforts of global health and addressing infectious disease threats, exhibited prominently by the trachoma epidemic caused by *Chlamydia trachomatis* [3,4], Zika virus, Yellow Fever, and EVD outbreaks [5,6,7]. The significant ophthalmic complications of the EVD epidemic further highlighted the key role that ophthalmologists may play in outbreak responses, particularly when ocular sequelae have been incompletely studied [8]. Given the potential for vision-compromising ophthalmic complications either during the acute phase of infectious illness disease or as long-term sequelae, as well as viral transmission to and from ocular surface mucosal surfaces, there is an urgent need to recognize and understand infectious eye diseases in the global health context.

We performed a computerized PubMed search for the journal articles cited in this review on 31 July 2020, which was updated on 1 March 2021 for currency. Search terms relevant to specific WHO High Priority pathogens of interest in this review (e.g., EVD, Nipah, Lassa fever) were combined with terms related to clinical disease, ophthalmic manifestations and treatment, and selected pathophysiologic mechanisms focusing on ocular manifestations. Ophthalmic search terms used included ophthalmic findings, ophthalmology, eye, ocular, retina, uveitis, conjunctiva, cornea, and retinopathy. For literature searches relevant to SARS-CoV-2, the names 2019-nCoV and COVID-19 were used. The literature search was performed by three authors for full review (SK, CLA, TF). The articles selected were further reviewed and relevant details were verified by ophthalmologist senior clinical researchers (JGS, SY). This review summarizes the key ophthalmic consequences of WHO High Priority pathogens, viral disease pathogenesis, disease findings, and areas of unmet research need (Table 1).

## 2. Pathophysiology of Ocular Manifestations of Viral Illness

The pathogenesis of ocular manifestations vary between viruses given different cell targets and disease-specific immunologic response. Respiratory viruses may often cause ocular symptoms and signs given the anatomic continuity between the ocular and respiratory systems via the nasolacrimal duct system. In addition, hand-eye contact increases the risk of contaminating the conjunctival epithelium from droplets and body fluids.

Delayed onset of some ocular symptoms may indicate a delayed immune response due to antigenic mimicry, delayed hypersensitivity reactions, or stimulation of a pathogenic lymphocyte reaction. Viral persistence in the eye for pathogens that may establish residence in the immune privileged eye may also contribute to inflammatory activation. In EVD survivors, for example, viral persistence has been detected and is associated with severe panuveitis, optic neuropathy, and iris heterochromia. Understanding this interplay between viral infection and severe inflammation is a key component in determination of an appropriate medical countermeasure, which may include antiviral and anti-inflammatory medications [8]. Besides direct lytic viral effects, inflammatory mechanisms and coagulation abnormalities can lead to endothelial damage which may lead to damage to the retinal circulation and resultant retinopathy [11,25]. Known pathophysiologic mechanisms specific for ocular disease are described in the subheadings for each viral pathogen.

## 3. Crimean-Congo Hemorrhagic Fever

Crimean-Congo hemorrhagic fever (CCHF) is an infection caused by the tick-borne Nairovirus. Regarding the origin of its name, the disease was first characterized in Crimea in 1944 and later in Congo in 1969. The virus presents initially as headache, high fever, back pain, joint pain, and vomiting. In addition, patients may exhibit red eyes, a flushed face, and petechiae on the palate. From days 4–14 of infection, illness progresses to severe bruising, nosebleeds, and uncontrolled bleeding. Fatality rate in hospitalized patients ranges from 9–50%. Long-term effects and complications have not been well-studied in survivors [10].

### Ocular Complications

One prior study characterized the ocular findings of patients with CCHF during an outbreak in Sivas, a city in the central Anatolia region of Turkey. From July through August 2017, 19 confirmed CCHF patients were evaluated and stratified into “severe” and “non-severe” groups. While patients did not report any visual complaints, ocular findings were observed in 14 of 19 patients (73.7%). Disease findings included multiple subconjunctival hemorrhages in seven patients (36.8%) and two patients with retinal hemorrhage (10.5%) All patients showed complete resolution after one month of follow-up. Prothrombin time was significantly different in patients with and without ocular findings [8,10].

## 4. Marburg Virus Disease

Marburg Virus is a zoonotic virus first discovered in 1967 when exposure to infected African green monkeys led to an outbreak of hemorrhagic fever [44]. The primary natural reservoir of the virus is thought to be the *Roussettus aegypticus*—The Egyptian fruit bat. The first phase of Marburg virus disease (MVD) presentation, the generalization phase, lasts approximately five days and is characterized by fever, chills, headache, and myalgia. Vomiting, nausea, diarrhea, conjunctivitis, and maculopapular rash have been identified as well. Five to thirteen days after the onset of the disease, the early organ phase, patients can present with severe dyspnea, exanthema, edema, and neurological symptoms including encephalitis, delirium, and irritability. Patients can experience visceral and mucosal hemorrhages as well as petechiae and hematemesis further into the stage. In rare instances, patients can progress to the late organ phase in which multiorgan failure and shock can ensue [44].

### Ocular Complications

Survivors of MVD can experience complications during convalescence, including ocular disease. During the 1975 Marburg outbreak, a 20-year-old nurse presented with complaints of malaise, myalgia, headache, and injected conjunctivae, followed by nausea, diarrhea, and a maculopapular rash characteristic of Marburg virus infection. Two months after being discharged after her symptoms had resolved, the patient developed severe pain in her right eye and blurry vision. Slit lamp examination showed white keratic precipitates, significant cell and flare in the anterior chamber, iritis, and a high intraocular pressure. She was diagnosed with active unilateral, hypertensive uveitis, and an aqueous tap confirmed Marburg viral persistence [13,14].

Recent work in MVD in a Rhesus macaque model has provided additional insights into ocular sequelae during acute MVD, as well as during convalescence. During acute MVD in this nonhuman primate (NHP) macaque model, inflammation of the iris of the eye, peripheral nerves and autonomic ganglia is observed. Marburg virus infection of the ciliary body of the eye and retina, testis, epididymis, ovary and mammary glands is also observed. Histopathology of the eye has shown macrophage infiltration of perivascular tissues of the eye and in situ hybridization has demonstrated Marburg virus genome within epithelial cells, smooth muscle, and stroma of the ciliary body [45]. These pathologic findings in the NHP model in combination with observations in MVD survivors provide insight into the potential mechanisms of ophthalmic disease, including viral persistence and associated inflammation.

## 5. Ebola Virus Disease

Ebola virus disease (EVD) is a zoonotic virus known to cause disease in humans and NHPs through close contact with infected animal reservoirs, with subsequent human–human transmission through direct contact with blood or body fluids. Over ten major EVD outbreaks have been documented since Ebola virus was first identified in simultaneous outbreaks in 1976 in South Sudan and the Democratic Republic of Congo. The largest EVD outbreak occurred from 2014–2016, resulting in over 11,000 deaths in Liberia, Guinea and Sierra Leone [8]. From May 2019 through June 2020, the Democratic Republic of Congo experienced the second largest documented Ebola outbreak with 3470 cases and a 66% case fatality rate [46]. This outbreak was marked by the successful implementation of an Ebola vaccine, and clinical trial that showed that two-antibody drugs, mAB114 and REGN-EB3, reduced mortality amongst hospitalized EVD patients [47]. However, containment of this outbreak was particularly complex given that it affected the North Kivu and Ituri provinces of the Democratic Republic of the Congo, a region challenged by political instability, ongoing conflict between armed militia groups, and targeted attacks on Ebola responders and health care facilities.

Affected individuals initially present after an incubation period of 2 to 21 days with fever, myalgia, and fatigue followed by vomiting, diarrhea, rash, and uncontrolled bleeding. Survivors can later suffer from post-Ebola virus disease syndrome with symptoms including arthralgias, abdominal pain, neurologic complications, sensorineural hearing loss, and ophthalmic complications [15].

### Ocular Complications

In the early stages of the disease, EVD patients can exhibit conjunctival injection, bilateral conjunctivitis, or subconjunctival hemorrhage. Uveitis and retinal disease have been described during the convalescent stage of EVD with disease ranging from mildly symptomatic to severe, sight-threatening inflammation without appropriate treatment [16]. Uveitic complications were initially observed in the 1995 Kikwit outbreak within Democratic Republic of the Congo where four EVD survivors developed uveitis 42–72 days post infection.

**Figure 1:**
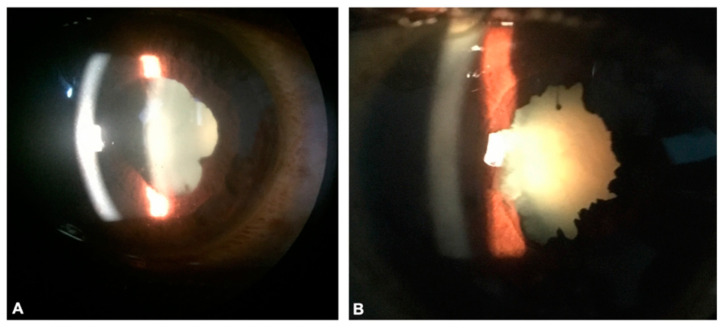
Anterior segment photographs of Ebola virus disease (EVD) survivors show keratic precipitates, posterior synechiae, and cataract due to untreated uveitis (**A**). High magnification of another EVD survivor shows diffuse posterior synechiae and dense cataractous lens which can develop from untreated uveitis or as a side effect of corticosteroid used to treat uveitis (**B**).

More recently in 2016, a study involving 96 EVD survivors in Liberia was conducted by our team in collaboration with Eternal Love Winning Africa (ELWA) Hospital and the Ministry of Health and Sanitation eye care providers. Uveitis, including anterior and posterior uveitis, was identified in 21 of the survivors, while EVD-associated optic neuropathy developed in three of the patients. Ocular examination showed the presence of keratic precipitates, anterior chamber cell, posterior synechiae, vitritis, and/or chorioretinal scars in patients with uveitis (Figure 1). In addition, ten of the EVD survivors had developed cataracts [15].

A prospective, controlled longitudinal natural history study in Liberia was reported by the National Institutes of Health-funded PREVAIL III Study Group in 2018, involving 966 Ebola virus antibody-positive survivors and 2350 antibody-negative close contacts. They reported a higher frequency of abnormal systemic findings and uveitis, with a prevalence of 24% in EVD survivors versus 12% in controls. The prevalence of uveitis increased over time in both study groups [17], indicating ongoing risk of disease burden in EVD survivors. These reports emphasize the importance of partnerships with in-country ophthalmology leadership during acute infectious outbreak settings. In addition, the high rate of ophthalmic disease observed is a reminder of the imperative to continue to understand the clinical and pathogenesis significance of ophthalmic disease findings for patient care amidst the acute care needs and survivor sequelae.

## 6. Human Coronavirus

Coronaviruses comprise a family of viruses first discovered to infect humans in 1969, which are transmitted primarily via respiratory droplets and direct mucous membrane contact. There are seven coronaviruses that infect humans [48] and were thought to primarily lead to benign, mild upper respiratory tract infections until the 2002 SARS-CoV (severe acute respiratory syndrome) outbreak that led to fatalities ranging from 0% to more than 50% depending on the age group affected. The global case fatality rate was estimated at approximately 11% [19]. Specifically, the SARS-CoV outbreak (i.e., now termed SARS-CoV-1), led to fatalities in over 770 people in more than 30 countries. Middle East respiratory syndrome coronavirus (MERS-CoV) was identified in Saudi Arabia in 2012. Thus far, over 2400 confirmed MERS cases have been documented with a case fatality rate of approximately 35% [20]. The ongoing Coronavirus-19 (COVID-19) pandemic due to SARS-CoV-2, which began in December 2019, has now led to over 108 million cases and 2.3 million deaths [18].

These highly transmissible pathogens result in lower respiratory tract infections that can rapidly progress to severe pneumonia. The basic reproductive rate (R_0_) of SARS-CoV-2 is estimated to be 2.5 (Range 1.8–3.6), which is thought to be higher than 2.0–3.0 for SARS-CoV (i.e., later reduced to 1.1 through control measures), and 0.69 for MERS-CoV. The case fatality rate increases steeply with age and is estimated at <1% for people aged 20–54 years, 1–5% in those aged 55–64 years, and 3–11% in individuals aged 65–84 years [22]. Unlike the other two diseases, the manifestations of COVID-19 from SARS-CoV-2 vary widely. While many patients develop respiratory disorders and experience fever, cough, and fatigue, some patients can have no respiratory symptoms at onset and exhibit extra-pulmonary manifestations, including headache, diarrhea, and vomiting.

### Ocular Complications

Conjunctivitis and ocular disease findings have not been previously reported in association with SARS or MERS. However, one study of health care workers with SARS-CoV showed that 3 of 36 patients (8%) had evidence of SARS-CoV RNA in their tear film [23,24].

Ocular involvement has been reported to a greater extent in association with COVID-19, although the global prevalence has greatly exceeded both SARS and MERS. Chemosis and conjunctivitis have been the most frequently reported ocular manifestations, with disease prevalence ranging from 2% to 32% [11,26]. Several studies have assessed the ocular surface for SARS-CoV-2 RNA utilizing conjunctival swab samples and Schirmer strip testing for tear film. While positive SARS-CoV-2 RNA testing has been documented in a minority of cases, its presence, particularly in association with conjunctivitis, emphasizes the importance of appropriate high-level safety protocols given the potential risk of transmission via tear film [49,50].

Retinal manifestations have also been described in limited case series and imaging studies. In one study of the retinal changes in 12 adults with COVID-19, all 12 patients showed hyperreflective lesions at the ganglion cell and inner plexiform layers on OCT, and four exhibited cotton wool spots and microhemorrhages [27]. Another study showed that 6 of 27 (22%) of COVID-19 positive patients with bilateral pneumonia showed cotton wool spots at a mean of 43 days following the onset of COVID-19 symptoms [28]. Interestingly, a recent study published found no evidence of retinal involvement in 43 patients hospitalized for COVID-19 pneumonia, although chorioretinitis of presumed opportunistic infection origin was observed [51].

## 7. Lassa Fever

Lassa fever is a severe viral hemorrhagic fever resulting from the Lassa virus, an Arenavirus transmitted to humans through direct contact with urine or feces from the *Mastomys* rodent species. Outbreaks of Lassa fever have mainly occurred in West African countries, including Sierra Leone, Guinea, Liberia, and Nigeria. The disease presents in four stages: individuals in stage 1 can experience weakness and high fever and can develop a sore throat with white patches, productive cough, and nausea or vomiting as they progress to stage 2. The later stages of the disease are characterized by more severe symptoms, including facial edema, convulsions, coma and death [29].

### Ocular Complications

Little is known about the ophthalmic manifestations of Lassa fever, but conjunctivitis has been observed in patients with acute disease. We recently reported the ophthalmic findings in a retrospective review of 31 Lassa fever survivors in the Kenema district, Sierra Leone [31]. Ophthalmic disease findings observed in Lassa fever survivors including cataract, chorioretinal scarring, retinal fibrosis and vitreous opacity. While median visual acuity was worse in Lassa survivors with ophthalmic manifestations, the potential association of these findings with Lassa virus requires further study. Interestingly, a recent study involving infected guinea pigs indicated the presence of the virus in the anterior segment of the eye [32]. Lassa virus immunostaining was prominent within the anterior segment filtration angle, ciliary body, and iris, particularly around vessels within the bulbar conjunctiva and peripheral cornea within animals with fatal infection.

## 8. Nipah Virus

Nipah Virus, a Paramyxoviridae virus, first emerged in Malaysia in 1998 and resulted in 265 cases of encephalitis and 105 deaths [33]. A more recent outbreak in 2018 in Kerala, India, resulted in 17 deaths [52]. The natural reservoir for the virus is the *Pteropus* bat, which sheds the virus through its saliva and urine. Thus, transmission to humans may occur when hunting bats or when consuming fruits contaminated by bats [53]. Individuals affected by Nipah Virus present with fever, headache, and vomiting, which can progress to respiratory illness and severe encephalitis associated with a high rate of mortality (32–92%) [33,34]. Neurologic characteristics include hypo- or areflexia, tachycardia, hypertension, meningismus, seizures, cerebellar signs, and segmental myoclonus [35].

### Ocular Complications

Ocular findings have been documented in the context of neurologic dysfunction and include pupillary abnormalities, oculomotor palsies, abnormal oculocephalic reflexes, and nystagmus [33,35,36]. Lim et al. described a longer term study in which five of 13 pig workers affected by Nipah virus encephalitis presented with visual symptoms, including persistent diplopia from a cranial nerve VI palsy and nystagmus. One patient experienced blurred vision after discharge from the hospital; further ocular examination, photography, and fluorescein angiography showed a branch retinal artery occlusion [37]. Another patient developed a left-sided Horner syndrome nine months after the outbreak with ptosis, miosis, and anhidrosis. Cerebrospinal fluid analysis showed IgG antibodies but not IgM antibodies and the Nipah virus culture was negative. While these delayed ophthalmic findings were attributed to be late manifestations of Nipah virus, the precise pathophysiology remains unknown.

## 9. Rift Valley Fever

Rift Valley Fever, caused by a phlebovirus, was first discovered in 1931 and caused a number of outbreaks across sub-Saharan Africa. The virus is transmitted to humans through direct contact with infected livestock. Rift Valley can cause a spectrum of disease, from mild symptoms such as fever and flu like-symptoms to severe encephalitis and bleeding in 10% of patients infected [38].

### Ocular Complications

Rift Valley Fever can lead to anterior and posterior segment complications with significant morbidity in up to 20% of patients. Many patients report blurred or decreased vision 1–3 weeks after symptom onset, and half of patients with macular lesions have permanent vision loss. Hazmi, et al. conducted a study involving infected individuals from the Rift Valley outbreak in Saudi Arabia in 2000. Two-hundred and twelve eyes from 143 patients were affected, including 47 eyes from 30 inpatients and 165 eyes from 113 outpatients. The interval between RVF onset and visual symptoms ranged from 4 to 15 days with a mean of 8.8 days. Interestingly, macular and paramacular retinitis was observed in all 212 eyes and a range of findings was observed. These included retinal hemorrhage, optic disc edema, and vasculitis. Visual acuity impairment at initial evaluation was poorer than 20/200 in 80% of eyes observed in the outpatient cohort. Moreover, non-granulomatous anterior uveitis, while transient, was also observed in association with RVF [39,40].

## 10. Mosquito-Transmitted Diseases

Chikungunya, Zika, and Dengue are additional diseases that pose major public health risks and were considered for the priority list. These illnesses often have similar clinical presentations with fever, headache, rash, and joint pain [25].

Chikungunya was first described in 1952 during an outbreak in Tanzania, and in 2004, the virus spread to more than 1/3 of the population during an outbreak in Kenya. Since then, Chikungunya has spread rapidly to over 60 countries. The joint pain associated with the disease is often debilitating and may last from a few days to years. It rarely progresses to a life-threatening illness; however, complications can include myocarditis, hepatitis, and other neurological and ocular disorders [54]. Ocular complications can present during or after resolution of systemic disease and can include photophobia, conjunctival injection, retroocular pain, and floaters. Anterior uveitis, optic neuritis, and retinitis are the most common complications with variable outcomes from full vision recovery to residual scotoma to loss of vision [25].

Zika virus is an arbovirus transmitted to humans by mosquitos with other modes of transmission documented including sexual, perinatal, and via blood transfusion. Zika virus was first identified in 1947 in Uganda and migrated to Asia in the 1940s. The largest outbreak was documented in 2015 in Brazil, infecting 0.4 to 1.3 million people. Approximately 75% of infected patients are asymptomatic, but infection can be associated with viral neurotropism and Guillain-Barre syndrome, causing subacute flaccid paralysis and death. Conjunctivitis, uveitis, and unilateral acute maculopathy have been reported in adults after acute infection. While congenital Zika syndrome initially associated with microcephaly as the leading finding in newborns, the 2015 outbreak raised awareness largely through work by Ventura et al., of visually significant macular scarring, retinal mottling, structural anomalies, chorioretinal atrophy, and optic nerve hypoplasia as complications. In one study of mothers with reported Zika virus infection symptoms, none of the mothers had ophthalmologic abnormalities, while 17 eyes of 10 children had ocular abnormalities [42].

Dengue virus remains asymptomatic in 75% of cases. However, Dengue contributes 500,000 hospitalizations annually, primarily in the Americas, Southeast Asia, and the Western Pacific. The typical clinical presentation of dengue fever, as well as mucosal bleeding, nausea, and vomiting, appears 3–14 days after infection. In severe cases, hemorrhagic disease can cause hypotension, shock syndrome, and multiple organ failure [43]. The pathogenesis of the reported ophthalmic manifestations is the same as systemic disease. Direct viral infection or activation of inflammation causes hemoconcentration, vasculitis, and coagulation disorders. While visual impairment from ocular disease is uncommon, cases of subconjunctival hemorrhage (37% of cases), uveitis, and maculopathy characterized by vasculitis and hemorrhage have all been reported [25].

## 11. Conclusions

As recent outbreaks including Zika and Ebola have highlighted the impact of epidemic and pandemic threats on vision health, there remains an imperative to advance diagnostic and therapeutic tools for ophthalmic care and research, as part of the public health responses during infectious disease outbreaks [15,16,17,42]. While ophthalmic findings have been observed in a minority of cases of COVID-19 (e.g., conjunctivitis, retinopathy), assessment of SARS-CoV-2 within the ocular surface has demonstrated the presence of SARS-CoV-2 viral RNA within tear film [11,27,28,50]. The protocols employed to understand the clinical phenotypes of these WHO high priority viral pathogens, as well as methods for ocular fluid sampling (i.e., tear film, aqueous humor), are key learnings that will inform research related to ophthalmic disease in other emerging infectious disease threats.

The ophthalmic sequelae and vision health consequences described in association with WHO High Priority Pathogens range in the degree of visual morbidity (i.e., high morbidity with Rift Valley Fever retinopathy [38,39] and potential for vision-threatening uveitis with EVD [8] versus infrequent conjunctivitis observed with COVID-19 [11]). However, there also remain public health implications given the unique ability for virus to persist within the eye (e.g., Marburg, Ebola virus) and potential for viral presence in tear film during acute infection (e.g., SARS-CoV-1 and SARS-CoV-2).

Understanding the disease phenotypes on this WHO high priority roadmap will provide not only useful information to for patients during epidemic and outbreak threats from known agents, but also will allow emergency responders to develop health systems needed to protect vision as outbreaks emerge and evolve in the future.

## Figures and Tables

**Table 1:** Summary of World Health Organization High Priority diseases with systemic and ophthalmic findings of clinical significance.

Disease	Virus Family	Geographic Region(s) and Countries Affected	Systemic Findings	Ophthalmic Disease Findings	Other Vision Health Implications
Crimean-Congo Hemorrhagic fever	Nairovirus *(Bunyavirus)*	Eastern Europe, Asia, Middle East, Central Africa, West Africa, South Africa, Madagascar. [9]	Headache, fever, back pain, joint pain, and vomiting. Illness progression to severe bruising, nosebleeds, and uncontrolled bleeding. [10]	Multiple subconjunctival hemorrhages, retinal hemorrhage. [10]	
Marburg-Virus Disease	Marburg Virus *(Filovirus)*	Central Africa—Democratic Republic of Congo, Angola; East Africa- Uganda, Kenya; South Africa, Germany, Netherlands, Serbia, United States, Yugoslavia. [9]	Fever, headache, malaise, diarrhea, vomiting. Extreme lethargy, and multiple organ failure as disease progresses without treatment. [11] Orchitis in severe cases. [12]	Acute MVD: Conjunctivitis, iritis, retinitis. [13] Convalescence: iritis, increased intraocular pressure, active unilateral hypertensive uveitis. [13,14]	Marburg viral persistence in aqueous tap during convalescence reported. [13,14]
Ebola Virus Disease	Ebola virus *(Filovirus)*	Democratic Republic of the Congo, West Africa—Sierra Leone, Liberia, Guinea, Nigeria, Senegal, Mali; United States, United Kingdom, Italy, Spain. [9]	Fever, vomiting, diarrhea, hypovolemic shock, and multi-organ failure without treatment. [15,16]	Acute EVD: Conjunctivitis, subconjunctival hemorrhage Convalescence: Anterior uveitis, Intermediate uveitis, panuveitis with heterochromia; uveitis complications if not treated; optic neuropathy. [16,17]	Risk of viral persistence in aqueous humor reported during convalescence. [16,17]
Human Coronavirus	*Coronaviridae*	SARS-CoV: Southeast Asia, Europe, South Africa. MERS-CoV: Middle East—Saudi Arabia, Lebanon, Iran, United Arab Emirates, Oman, Jordan; Europe- Greece, Germany, Italy; Asia; Philippines, Malaysia, China, Thailand; United States, United Kingdom. [9] SARS-CoV-2: 191 countries affected globally to date. [18]	SARS-CoV: Fever, cough, headache, malaise, shortness of breath. [18,19] MERS-CoV: fever, cough, shortness of breath, diarrhea, vomiting [20,21] SARS-CoV-2: Fever, cough, fatigue, headache, shortness of breath, sore throat, loss of taste or smell. [18,22]	SARS-CoV: SARS-CoV RNA seen in tear film during early phase of SARS infection. [23,24] MERS-CoV: No reports of ophthalmic findings to-date SARS-CoV-2: Chemosis and Conjunctivitis reported in COVID-19 infection, SARS-CoV-2 RNA seen in conjunctival swab and Schirmer’s strip. [25,26] Retinal manifestations including hyperreflective lesions reported on OCT, cotton wool spots and microhemorrhages seen. [27,28]	
Lassa fever	Mammarenavirus *(Arenavirus)*	West Africa- Sierra Leone, Guinea, Liberia, and Nigeria. [9]	Fever, sore throat, vomiting, malaise. Neurological complications including hearing loss in severe cases. [29,30]	Acute Lassa fever: Conjunctivitis Convalescence: cataract, chorioretinal scarring, retinal fibrosis, and vitreous opacity noticed. Anterior uveitis, iritis. [31,32]	Visual acuity worsened in LHF survivors with ophthalmic manifestations. [32]
Nipah Virus	Henipavirus *(Paramyxoviridae)*	Malaysia, Bangladesh, India, Singapore, Cambodia, Ghana, Indonesia, Madagascar, the Philippines, and Thailand. [9]	Fever, headache, vomiting. Respiratory and neurologic complications including seizures recorded as illness progresses. [33,34]	Neurological associated dysfunctions including pupillary abnormalities, oculomotor palsies, abnormal oculocephalic reflexes, nystagmus, persistent diplopia from cranial nerve VI palsy, and retinal artery occlusion. [35,36]	Ptosis, miosis, and anhidrosis associated with Horner syndrome noticed as late manifestations. [37]
Rift Valley fever	Phlebovirus *(Bunyaviridae)*	Sub-Saharan Africa-Egypt, the Gambia, Kenya, Madagascar, Mauritania, Mozambique, Namibia, Saudi Arabia, Senegal, South Africa, South Sudan, Sudan, Tanzania, Yemen, Zambia, Zimbabwe. [25]	Fever, malaise, back pain, and dizziness. Neurological complications including seizures as disease progresses. [38]	Non-granulomatous anterior uveitis, Macular and paramacular retinitis, retinal hemorrhage, optic disc edema, vasculitis. [39,40]	
Mosquito transmitted diseases	Chikungunya: Alphavirus *(Togavirus)* Zika and Dengue: Flavivirus	Chikungunya: Sub-Saharan Africa- Tanzania, Kenya; India, Indonesia, Thailand, Brazil, Colombia. [9] Zika: South America, Central America, North America, and the Caribbean. [9] Dengue: Caribbean- Guyana, Grenada, Haiti, Jamaica, Panama; Asia- Bangladesh, Cambodia, Malaysia, Thailand; Africa- Ethiopia, Burkina Faso, Eritrea, Somalia. [41]	Chikungunya: fever, headache, joint pain, joint swelling. [25] Zika: Asymptomatic in most cases. Mild symptoms including fever, rash, headache, and joint pain seen in some cases [42]. Dengue: asymptomatic in most cases. Mucousal bleeding, nausea and vomiting in some cases. Multiple organ failure and shock seen as complications if left untreated. [43]	Chikungunya: photophobia, conjunctival injection, retroocular pain, and floaters. Anterior uveitis, optic neuritis, and retinitis. [25] Zika: Conjunctivitis, Uveitis, unilateral acute maculopathy noticed in adults during acute phase. Macular scarring, retinal mottling, chorioretinal atrophy, optic nerve hypoplasia noticed as complications in congenital syndrome. [42] Dengue: Uveitis, Sub-conjunctival hemorrhage, maculopathy. [25]	

## Data Availability

Sources of data used for this review have been fully referenced.

## References

[1] Røttingen J.A., Gouglas D., Feinberg M., Plotkin S., Raghavan K.V., Witty A., Draghia-Akli R., Stoffels P., Piot P. (2017). New Vaccines against Epidemic Infectious Diseases. N. Engl. J. Med..

[2] Prioritizing Diseases for Research and Development in Emergency Contexts. https://www.who.int/activities/prioritizing-diseases-for-research-and-development-in-emergency-contexts.

[3] Feibel R.M. (2011). Fred Loe, MD, and the history of trachoma. Arch. Ophthalmol..

[4] Hu V.H., Harding-Esch E.M., Burton M.J., Bailey R.L., Kadimpeul J., Mabey D.C.W. (2010). Epidemiology and control of trachoma: Systematic review. Trop. Med. Int. Health.

[5] Brandão-de-Resende C., Cunha L.H.M., Oliveira S.L., Pereira L.S., Oliveira J.G.F., Santos T.A., Vasconcelos-Santos D.V. (2019). Characterization of retinopathy among patients with Yellow Fever during 2 outbreaks in Southeastern Brazil. JAMA Ophthalmol..

[6] Ventura C.V., Maia M., Bravo-Filho V., Góis A.L., Belfort R. (2016). Zika virus in Brazil and macular atrophy in a child with microcephaly. Lancet.

[7] Shantha J.G., Yeh S., Acharya N. (2019). Insights from 2 outbreaks in southeastern Brazil: Yellow Fever retinopathy. JAMA Ophthalmol..

[8] Shantha J.G., Crozier I., Yeh S. (2017). An update on ocular complications of Ebola virus disease. Curr. Opin. Ophthalmol..

[9] World Health Organization Emergencies-Disease Outbreaks. https://www.who.int/emergencies/diseases/en/.

[10] Engin A., Erdogan H., Ozec A.V., Elaldi N., Toker M.I., Bakir M., Dokmetas I., Arici M.K. (2009). Ocular findings in patients with Crimean-Congo hemorrhagic fever. Am. J. Ophthalmol..

[11] Wu P., Duan F., Luo C., Liu Q., Qu X., Liang L., Wu K. (2020). Characteristics of Ocular Findings of Patients with Coronavirus Disease 2019 (COVID-19) in Hubei Province, China. JAMA Ophthalmol..

[12] World Health Organization Marburg Virus Disease. https://www.who.int/health-topics/marburg-virus-disease/#tab=tab_2].

[13] Gear J.S., Cassel G.A., Gear A.J., Trappler B., Clausen L., Meyers A.M., Kew M.C., Bothwell T.H., Sher R., Miller G.B. (1975). Outbreake of Marburg virus disease in Johannesburg. BMJ.

[14] Kuming B.S., Kokoris N., Spalton D.J., Palmer S., Logan L.C. (1977). Uveal involvement in Marburg virus disease. Br. J. Ophthalmol..

[15] Shantha J.G., Crozier I., Hayek B.R., Bruce B.B., Gargu C., Brown J., Fankhauser J., Yeh S. (2017). Ophthalmic Manifestations and Causes of Vision Impairment in Ebola Virus Disease Survivors in Monrovia, Liberia. Ophthalmology.

[16] Connors D.B., Shantha J.G., Yeh S. (2015). Emerging Causes of Viral-associated Uveitis. Int. Ophthalmol. Clin..

[17] Sneller M.C., Reilly C., Badio M., Bishop R.J., Eghrari A.O., Moses S.J., Johnson K.L., Gayedyu-Dennis D., Hensley L.E., Higgs E.S. (2019). A Longitudinal Study of Ebola Sequelae in Liberia. N. Engl. J. Med..

[18] COVID-19 Dashboard by the Center for Systems Science and Engineering (CSSE) at Johns Hopkins University. https://gisanddata.maps.arcgis.com/apps/opsdashboard/index.html#/bda7594740fd40299423467b48e9ecf6.

[19] World Health Organization Consensus Document on the Epidemiology of Severe Acute Respiratory Syndrome (SARS). https://www.who.int/csr/sars/en/WHOconsensus.pdf.

[20] Killerby M.E., Biggs H.M., Midgley C.M., Gerber S.I., Watson J.T. (2020). Middle East Respiratory Syndrome Coronavirus Transmission. Emerg. Infect. Dis..

[21] World Health Organization Middle East Respiratory Syndrome Coronavirus (MERS-CoV). https://www.who.int/news-room/fact-sheets/detail/middle-east-respiratory-syndrome-coronavirus-(mers-cov).

[22] Petersen E., Koopmans M., Go U., Hamer D.H., Petrosillo N., Castelli F., Storgaard M., Al Khalili S., Simonsen L. (2020). Comparing SARS-CoV-2 with SARS-CoV and influenza pandemics. Lancet Infect. Dis..

[23] Loon S.-C., Teoh S.C.B., Oon L.L.E., Se-Thoe S.-Y., Ling A.-E., Leo Y.-S., Leong H.-N. (2004). The severe acute respiratory syndrome coronavirus in tears. Br. J. Ophthalmol..

[24] Kennedy A., Shantha J.G., Li J.-P.O., Faia L.J., Hartley C., Kuthyar S., Albini T.A., Wu H., Chodosh J., Ting D.S. (2020). SARS-CoV-2 and the Eye: Implications for the Retina Specialist from Human Coronavirus Outbreaks and Animal Models. J. Vitr. Dis..

[25] de Andrade G.C., Ventura C.V., Mello Filho P.A.d.A., Maia M., Vianello S., Rodrigues E.B. (2017). Arboviruses and the eye. Int. J. Retin. Vitr..

[26] Zhang X., Chen X., Chen L., Deng C., Zou X., Liu W., Yu H., Chen B., Sun X. (2020). The evidence of SARS-CoV-2 infection on ocular surface. Ocul. Surf..

[27] Landecho M.F., Yuste J.R., Gándara E., Sunsundegui P., Quiroga J., Alcaide A.B., García-Layana A. (2020). COVID-19 retinal microangiopathy as an in vivo biomarker of systemic vascular disease?. J. Int. Med..

[28] Marinho P.M., Marcos A.A., Romano A.C., Nascimento H., Belfort R. (2020). Retinal findings in patients with COVID-19. Lancet.

[29] Richmond J.K., Baglole D.J. (2003). Lassa fever: Epidemiology, clinical features, and social consequences. BMJ.

[30] Centers for Disease Control and Prevention (CDC) Lassa Fever-Sign and Symptoms. https://www.cdc.gov/vhf/lassa/symptoms/index.html.

[31] Li A.L., Grant D., Gbakie M., Kanneh L., Mustafa I., Bond N., Engel E., Schieffelin J., Vandy M.J., Yeh S. (2020). Ophthalmic manifestations and vision impairment in Lassa fever survivors. PLoS ONE.

[32] Gary J.M., Welch S.R., Ritter J.M., Coleman-McCray J., Huynh T., Kainulainen M.H., Bollweg B.C., Parihar V., Nichol S.T., Zaki S.R. (2019). Lassa Virus Targeting of Anterior Uvea and Endothelium of Cornea and Conjunctiva in Eye of Guinea Pig Model. Emerg. Infect. Dis..

[33] Ang B.S.P., Lim T.C.C., Wang L. (2018). Nipah Virus Infection. J. Clin. Microbiol..

[34] Shariff M. (2019). Nipah virus infection: A review. Epidemiol. Infect..

[35] Goh K.J., Tan C.T., Chew N.K., Tan P.S., Kamarulzaman A., Sarji S.A., Wong K.T., Abdullah B.J., Chua K.B., Lam S.K. (2000). Clinical features of Nipah virus encephalitis among pig farmers in Malaysia. N. Engl. J. Med..

[36] Banerjee S., Gupta N., Kodan P., Mittal A., Ray Y., Nischal N., Soneja M., Biswas A., Wig N. (2019). Nipah virus disease: A rare and intractable disease. Intract. Rare Dis. Res..

[37] Lim C.C.T., Lee W.L., Leo Y.S., Lee K.E., Chan K.P., Ling A.E., Oh H., Auchus A.P., Paton N.I., Hui F. (2003). Late clinical and magnetic resonance imaging follow up of Nipah virus infection. J. Neurol. Neurosurg. Psychiatry.

[38] Hartman A. (2017). Rift Valley Fever. Clin. Lab. Med..

[39] Al-Hazmi A., Al-Rajhi A.A., Abboud E.B., Ayoola E.A., Al-Hazmi M., Saadi R., Ahmed N. (2005). Ocular complications of Rift Valley fever outbreak in Saudi Arabia. Ophthalmology.

[40] Agarwal A., Kumar A., Khairallah M., Abboud E., Gupta V., Nguyen Q.D., LeHoang P., Herbort C.P. (2017). Rift Valley Fever Uveitis. The Uveitis Atlas.

[41] World Health Organization (WHO) Ebola Virus Disease. https://www.who.int/news-room/fact-sheets/detail/ebola-virus-disease.

[42] de Paula Freitas B., Ventura C.V., Maia M., Belfort R. (2017). Zika virus and the eye. Curr. Opin. Ophthalmol..

[43] Ng A.W., Teoh S.C. (2015). Dengue eye disease. Surv. Ophthalmol..

[44] Mehedi M., Groseth A., Feldmann H., Ebihara H. (2011). Clinical aspects of Marburg hemorrhagic fever. Future Virol..

[45] Cooper T.K., Sword J., Johnson J.C., Bonilla A., Hart R., Liu D.X., Bernbaum J.G., Cooper K., Jahrling P.B., Hensley L.E. (2018). New Insights into Marburg Virus Disease Pathogenesis in the Rhesus Macaque Model. J. Infect. Dis..

[46] Ilunga Kalenga O., Moeti M., Sparrow A., Nguyen V.K., Lucey D., Ghebreyesus T.A. (2019). The Ongoing Ebola Epidemic in the Democratic Republic of Congo, 2018–2019. N. Engl. J. Med..

[47] Maxmen A. (2020). Second-deadliest Ebola outbreak ends in Democratic Republic of the Congo. Nat. Cell Biol..

[48] Coronavirus. https://www.cdc.gov/coronavirus/types.html.

[49] Wu X.N., Lightman S., Tomkins-Netzer O. (2019). Viral retinitis: Diagnosis and management in the era of biologic immunosuppression: A review. Clin. Exp. Ophthalmol..

[50] Chen L., Liu M., Zhang Z., Qiao K., Huang T., Chen M., Xin N., Huang Z., Liu L., Zhang G. (2020). Ocular manifestations of a hospitalised patient with confirmed 2019 novel coronavirus disease. Br. J. Ophthalmol..

[51] Pirraglia M.P., Ceccarelli G., Cerini A., Visioli G., D’Ettorre G., Mastroianni C.M., Pugliese F., Lambiase A., Gharbiya M. (2020). Retinal involvement and ocular findings in COVID-19 pneumonia patients. Sci. Rep..

[52] Nipah Virus-India World Health Organization. https://www.who.int/csr/don/07-august-2018-nipah-virus-india/en/.

[53] Gurley E.S., Hegde S.T., Hossain K., Sazzad H.M., Hossain M.J., Rahman M., Sharker M.Y., Al. E.S.G.E., Islam M.S., Epstein J.H. (2017). Convergence of Humans, Bats, Trees, and Culture in Nipah Virus Transmission, Bangladesh. Emerg. Infect. Dis..

[54] Martínez-Pulgarin D.F., Chowdhury F.R., Villamil-Gómez W.E., Rodriguez-Morales A.J., Blohm G.M., Paniz-Mondolfi A.E. (2016). Ophthalmologic aspects of chikungunya infection. Travel Med. Infect. Dis..

